# Recent advances in etiology and treatment of von Hippel-Lindau Disease (VHLD)

**DOI:** 10.1007/s10555-026-10370-x

**Published:** 2026-08-13

**Authors:** Shabnam Pirestani, Emmanuelle Nicolas, Robert L. Broadrup, Margie L. Clapper, Erica Golemis

**Affiliations:** 1 Program in Cancer Signaling and Microenvironment Fox Chase Cancer Center, Philadelphia, PA 19111 USA; 2 Drexel University College of Medicine, Philadelphia, PA 19102 USA; 3 Department of Cancer and Cellular Biology, Lewis Katz School of Medicine at Temple University, 3500 North Broad St., Philadelphia, PA 19140 USA; 4 Program in Cancer Prevention and Control, Fox Chase Cancer Center, Philadelphia, PA 19111 USA

**Keywords:** Targeted therapy, Clinical trial, Kidney cancer, Hypomorphic mutation, VEGF

## Abstract

Von Hippel-Lindau disease (VHLD) is a rare autosomal dominant disease, occuring in ~ 1 in 35,000 individuals. Overall, individuals with inherited mutations in the *VHL* tumor suppressor gene are predisposed to a variety of cancer, including high frequencies of clear cell renal cell carcinoma (ccRCC), pancreatic neuroendocrine tumors, and hemangioblastomas, as well as benign cystic conditions and other cancers. The degree of risk for each of these pathological conditions depends on the location and severity of the inherited germline mutation, and the specific VHL protein interactions and functions disrupted. A core VHL protein function is as the targeting subunit of an E3 ligase complex, with protein degradation activity based on interactions with elongins (ELOB, ELOC), Cullin 2 (CUL2), and RBX1. For ccRCC and some other cancers, loss of VHL-dependent degradation of key substrates—the transcription factors hypoxia-inducible factor alpha (HIF-1α and HIF-2α)—and upregulation of HIF-dependent transcripts are critical to promote tumor formation. For this reason, drugs such as the HIF signaling inhibitor belzutifan have emerged as promising clinical agents for treatment of VHLD patients prone to ccRCC. However, other biological consequences of VHL loss are independent of HIFα degradation, and in some cases independent of the VHL ubiquitin ligase activity. Non-canonical activities of VHL include regulation of microtubule stability, mitotic progression, and ciliation, as well as formation of the extracellular matrix (ECM); the degree to which disruption of these activities contributes to VHLD is currently not well understood. This review provides a concise update of the current literature on VHLD pathogenesis, the relationship of VHL structure and protein interactions to the spectrum of phenotypes associated with VHLD, and current and proposed treatment, prevention, and interception of cancer formation for VHLD patients.

## Introduction: pathogenesis of von Hippel-Lindau Disease (VHLD)

**Fig. 1:**
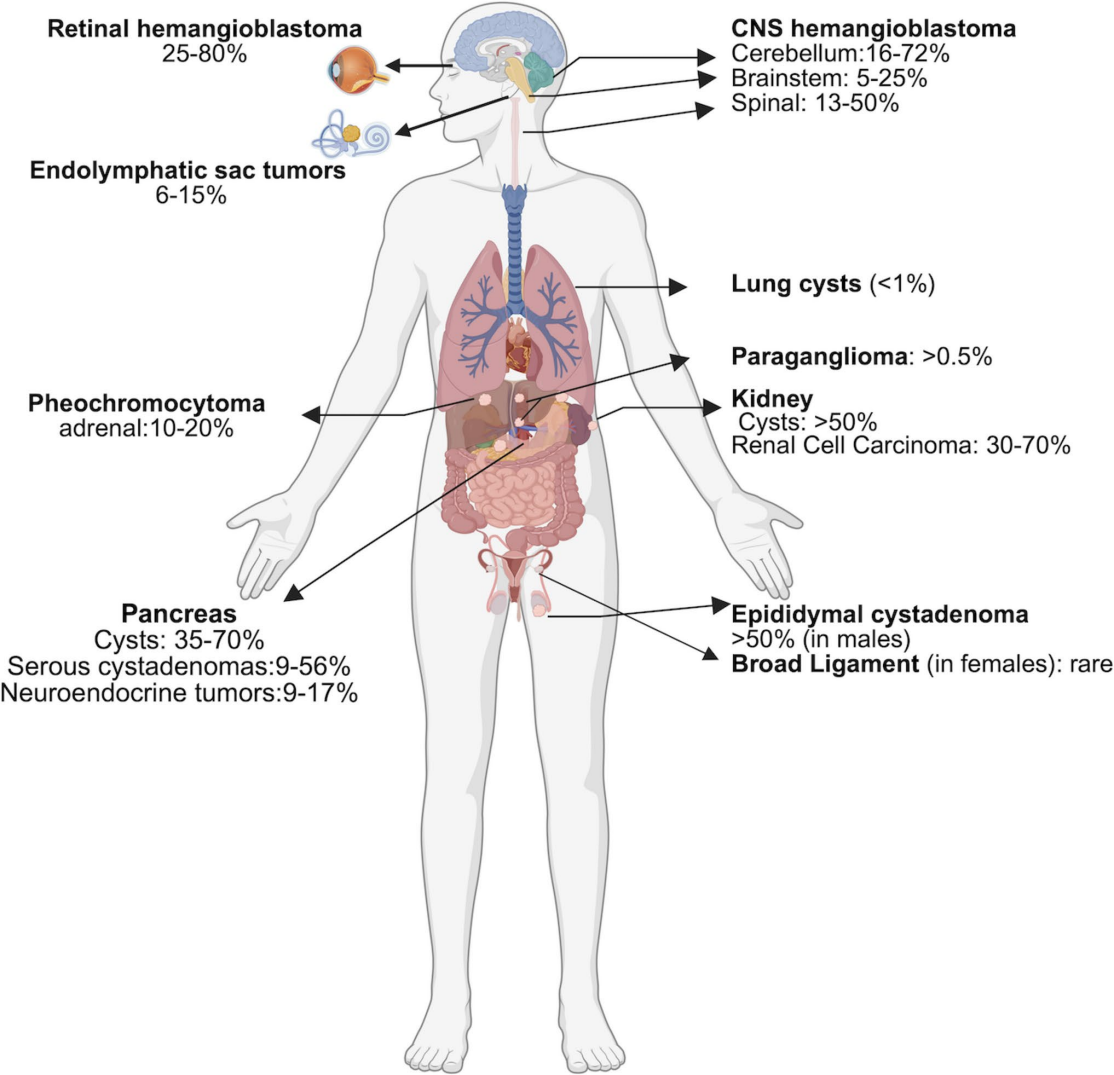
VHLD-associated pathologies. Schematic diagram listing the incidence of noted diseases occurring among individuals bearing germline mutations in VHL. Likelihood of a specific tumor type is associated with sub-type of inherited VHL mutation

VHLD is a rare autosomal dominant disease that has been reported to affect as many as 1 in 35,000 individuals globally [1, 2], and places affected individuals at high risk of cancer and other pathological conditions, with very high penetrance [1]. VHLD arises in individuals who inherit a damaged allele of the *VHL* tumor suppressor gene; approximately 50% of the known VHLD gene variants represent missense mutations [3]. When the second copy of *VHL* is inactivated somatically, individuals with VHLD are prone to develop cancers, with clear cell renal cell carcinoma (ccRCC) the most common, followed by pancreatic neuroendocrine tumors and various tumors of the nervous system (e.g. paragangliomas) and the vascular system (e.g. hemangioblastomas), as well as additional benign pathological conditions such as cystic formation (Fig. 1). The complex presentation of this syndrome, which can include recurrent formation of primary tumors, requires complex clinical management and poses a significant economic burden [4, 5].

The presentation of VHLD is not uniform, as discrete germline variants in VHL predispose individuals to distinct spectrums of risk for development of specific forms of cancer and other pathological conditions. Mutations that result in total loss of VHL protein (pVHL) function (whole-gene deletions, nonsense variants, and frameshifting insertions and deletions) typically present as type 1 VHLD, characterized by a high risk of ccRCC and hemangioblastoma and low risk of paraganglioma. In contrast, while many missense mutations and moderately destabilizing mutations are predisposing to type 1 VHLD, such mutations are often preferentially associated with various forms of type 2 VHLD. These present with high (type 2B), moderate (type 2 A), or low (type 2 C) risk of forming ccRCC [6, 7] but frequently lead to pheochromocytoma, hemangioblastoma, and other tumor types (Fig. 1). Among missense mutations, the specific location within the pVHL protein structure, differentially impacts critical protein interactions and significantly affects the disease spectrum, as discussed below.

Improvements in the accuracy of genetic counseling for individuals and families with *VHL* variants linked to VHLD, and the development of therapies for VHLD, both require a detailed understanding of the functional consequences of unique changes in the VHL protein. In addition, although VHLD is rare, we note that disruption of *VHL* by somatic mutation, hypermethylation of its promoter, or chromosomal loss, is also seen in the majority of cases of sporadic ccRCC [1]. Over 434,00 individuals globally are diagnosed with sporadic ccRCC each year, with ccRCC representing 70–80% of cases; survival is poor when the cancer is identified at an advanced stage [8]. Together, these findings have motivated extensive efforts to develop therapies targeting pVHL and its effectors for ccRCC [9]. We discuss the current state of development of therapies for use in prevention, interception, or treatment of VHLD.

### Biological activities of VHL: canonical functions in proliferation, metabolism, and angiogenesis regulated through hypoxia inducible factors (HIFs)

**Fig. 2:**
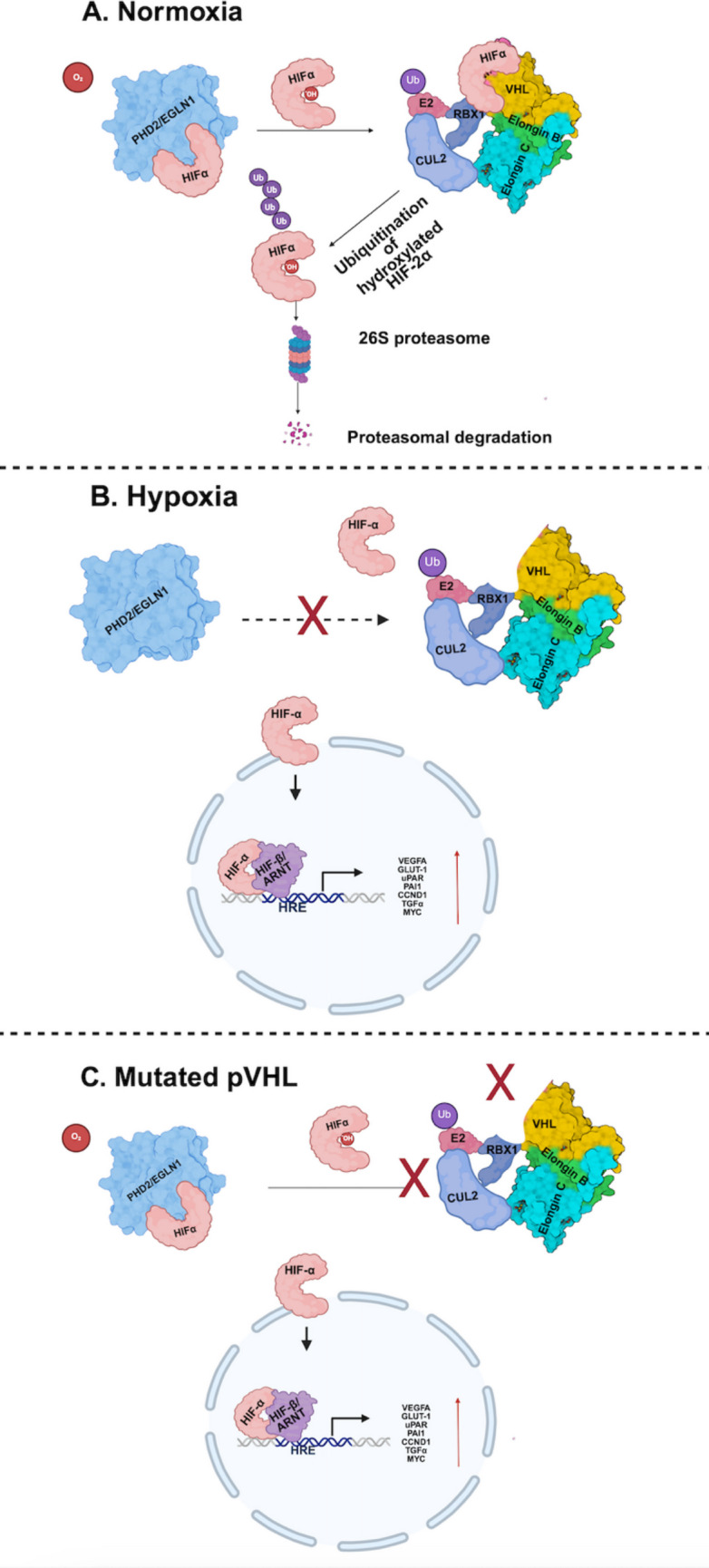
pVHL function in control of HIFα signaling. Representation of relationship between wild type VHL and HIFα in control of transcription under conditions of normoxia (**A**) or hypoxia (**B**), or in the context of a VHL mutation (**C**)

Loss of VHL leads to both tumor-intrinsic and tumor-extrinsic biological consequences, including increased proliferative index, reduced sensitivity to hypoxic versus normoxic growth conditions, epithelial-mesenchymal transition (EMT), altered metabolism, increased invasion through extracellular matrix (ECM), increased intratumoral angiogenesis, and a pro-inflammatory tumor microenvironment [10–12]. Mechanistically, the most highly validated function of pVHL as a tumor suppressor is as a targeting component of a ubiquitin ligase (Fig. 2). Specifically, VHL is the substrate-targeting subunit of a Skp1-cullin-F-box (SCF-2) E3 ubiquitin ligase complex, which consists of Elongins B and C (ELOB/ELOC), Cullin-2 (CUL2), and RBX1 [1]. In ccRCC formation, the most important substrates targeted by VHL for ubiquitin-mediated degradation are members of the hypoxia-inducible factor (HIF) family of transcription factors, which includes HIF-1α and HIF-2α [1]. pVHL recognition and degradation of HIFα proteins depend on the latter bearing a hydroxyproline post-translational modification in response to a normoxic environment (high O_2_) (Fig. 2A); under hypoxic conditions, this modification does not occur, leading to elevated HIF expression (Fig. 2B). The majority of cancer-causing VHL mutations, and all VHL mutations associated with ccRCC, result in loss of the ability to degrade HIFα proteins with upregulation of HIF-2α strongly correlated, in particular, with oncogenic transformation [13, 14] (Fig. 2C).

Hypoxia induces expression of both HIF-1α and HIF-2α; these two structurally related proteins then heterodimerize with additional consitutively expressed proteins, ARNT and ARNT2 (also known as HIF-1β and HIF-2β). While both HIFa can bind with both ARNT and ARNT2, ARNT is the predominant partner, with ARNT2 having a more tissue-specific expression. The HIF-1α/ARNT and HIF-2α/ARNT heterodimers bind overlapping but non-identical transcriptional targets. Compensatory responses to transient hypoxia rely most heavily on HIF-1α, which is induced rapidly and transcribes genes that enhance glycolysis and suppress oxidative phosphorylation [15]. In contrast, viability during longer-term exposure to hypoxia, or in the case of absent or significantly impaired VHL, depends more on the function of HIF-2α, which reprograms cells to increase lipid storage and reduce the metabolism of fatty acids and amino acids [16] (discussed in detail in [17]).

Some of the earliest identified HIF-regulated genes and processes implicated in the formation of ccRCC (Fig. 2) include VEGFA, which promotes angiogenesis; glucose transporter-1 (GLUT-1), which increases glucose uptake to alter metabolism; and urokinase-type plasminogen activator receptor (uPAR) and plasminogen activator inhibitor-1 (PAI1), which contribute to remodeling of the extracellular matrix, angiogenesis, and tumor invasion [18]. Other important HIF-regulated transcripts include cyclin D1 (CCND1), which promotes cell cycle progression [19], and TGFα, which activates epidermal growth factor receptor to promote cell growth [19]. In some cases, as with the oncogene and globally acting transcription factor MYC, HIF proteins promote MYC activity by directly associating with MYC or MYC complexes to enhance their action instead of increasing transcription [20, 21]. Besides increasing proliferation, MYC participates in the reorganization of tumor metabolism by contributing to the characteristic accumulation of lipid droplets in ccRCC [22]. HIF-regulated targets that promote ccRCC have been reviewed recently in detail [10].

Importantly, a recent saturating mutagenesis study using CRISPR/Cas9 gene editing examined 2,268 single nucleotide variants (SNVs) in VHL that spanned the protein sequence. This work identified a close correlation between mutations annotated as pathogenic for ccRCC and loss of the ability of pVHL bearing these mutations to negatively regulate HIF proteins [23]. This emphasized the essential role of HIF elimination as a critical pVHL target for formation of aggressive cancers. This conclusion is strongly bolstered by the great success of the HIF-2α/heterodimerization inhibitor belzutifan in treating VHL-mutated ccRCC, as discussed below.

### Structure–function relations of VHL, HIF, and elongins

The *VHL* gene was first cloned in 1993, following extensive positional mapping efforts involving multiple research teams [24]. *VHL* expresses multiple splice variants. The two major pVHL protein isoforms encoded by *VHL* include a 213-amino acid (a.a.) isoform known as pVHL30 (pVHL_1–213_, referred to herein as pVHL) and a 160-a.a. isoform, pVHL19 (pVHL_54–213_) [25, 26]. pVHL19 arises from an internal alternative translation initiation (beginning at Met54 within the open reading frame of pVHL30), which eliminates an N-terminal residues 1–53. Both isoforms display tumor suppressor abilities [25, 26], emphasizing the importance of the carboxy-terminal domains of the protein for critical functions and protein interactions.

**Fig. 3:**
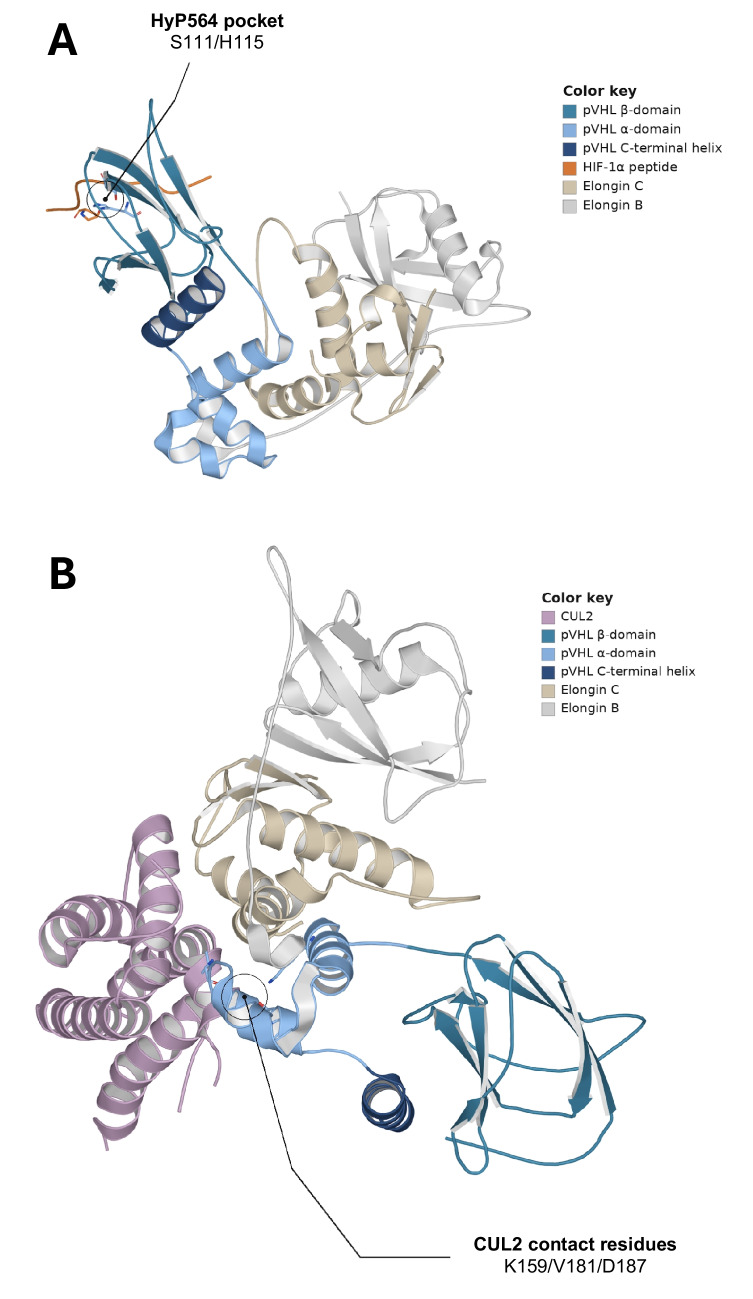
Structure of VHL-HIF-elongin interactions. **A**. The HIF-1α peptide-bound VCB core complex (based on PDB 1LM8). pVHL is colored by residue-defined regions: β-domain, residues 63–154; α-domain, residues 155–192; and modeled C-terminal helix, residues 193–207. The HIF-1α peptide is shown in orange, Elongin C in tan, and Elongin B in gray. The hydroxyproline (Hyp) recognition site is noted, illustrating contact between HIF-1α Hyp564 and pVHL S111 and H115. **B**. The VHL–EloB–EloC–CUL2 interface. The same residue-defined pVHL coloring is used as in (**A**). CUL2 is shown in mauve, Elongin C in tan, and Elongin B in gray. The pVHL-CUL2 contact residues (K159, V181, and D187) are highlighted, with interaction described in the text

The structure of VHL was first addressed in a number of landmark papers [27–29] that also probed VHL interactions with key partners (Fig. 3**)**. The complex of VHL bound to elongins B and C (the core VCB E3 ligase complex) was solved based on the pVHL19 isoform. This work identified an N-terminal region of approximately 100 amino acids containing seven β-strands arranged as a β-sandwich, designated the β-domain (residues 63–154 of the complete pVHL sequence), and a shorter C-terminal region containing α-helices, with the domains brought into proximity by two linker domains and a polar interface. Three of the α-helices were designated as the α-domain (residues 155–192); the final modeled C-terminal helix, approximately residues 193–207 in the deposited structures, packs against the β-domain [27]. Primary contacts between VHL and the elongins are based on association between the VHL α-domain and α-helices of elongin C, packing as a 4-helix cluster held together largely by hydrophobic interactions. Later work [30, 31] performed with the full length pVHL30 added the E3 ligase CUL2 and RBX1 to the VCB complex, and revealed a direct interaction between VHL and CUL2 involving contact sites in the pVHL α-domain, with VHL residues K159 and D187 mediating hydrogen bonding and salt bridge interactions, and V181 making an additional hydrophobic contact. Biochemical studies indicate that association with this complex stabilizes intracellular pVHL [32].

The structure of pVHL bound to full-length HIF-α substrates has not been solved. However, several crystal structures have defined the interaction of the VHL–Elongin B/C complex with short hydroxylated HIF degron peptides from HIF-1α or HIF-2α [28, 29, 33]. The HIF-1α structures include peptides centered on the C-terminal oxygen-dependent degradation domain containing hydroxylated Pro564; the second HIF-1α degron centered on Pro402 is also important for pVHL recognition but is not represented in these VHL-bound crystal structures. The analogous HIF-2α structures contain hydroxylated Pro531. These structures show that both HIF-1α and HIF-2α degron peptides bind the pVHL β-domain, adopting an extended conformation along a shallow, partially exposed groove on the β-sandwich. The peptide makes multiple main-chain/main-chain contacts with pVHL, while the hydroxyproline side chain inserts into a conserved pocket formed by residues including W88, Y98, S111, H115, and W117. In this pocket, the hydroxyproline 4-hydroxyl group satisfies buried hydrogen-bonding interactions with S111 and H115; without hydroxylation, burial of these polar groups would be energetically unfavorable. Residues surrounding the HIF-binding site are highly conserved, and structure–function studies have shown that mutations affecting this surface, including Y98, S111, and W117, disrupt pVHL–HIF interaction [34].

### Targeting HIF signaling for VHL-linked pathology

Development of therapeutic agents to treat tumors with mutated VHL have typically focused on the HIF-VEGF axis, and were developed to treat sporadic ccRCC. The earliest approaches focused on inhibition of VEGF or its receptor VEGFR/KDR, and included antibodies and small molecule inhibitors. A number of such agents have been approved and provide significant clinical benefits for patients with ccRCC and some other forms of cancer [35]. Unfortunately, these agents have significant side effects (defects in wound healing, blood clotting, and hypertension) that make them inappropriate for the long-term application that would be required for interception of nascent cancers in non-symptomatic individuals with VHLD.

Recent development of drugs that target HIF2 interactions have transformed the treatment of ccRCC. Successful creation of HIF2 inhibitors was based on the use of computational approaches to identify a surface pocket on HIF-2α. This was coupled with design of compounds that bound the pocket and created an allosteric effect that disrupted the ability of HIF-2α to bind its partner protein, ARNT, and therefore kept it from binding DNA [36, 37]. A number of drugs have been developed based on this paradigm. The first-in-class compound belzutifan (also known as MK-6482 and previously designated PT2977) was approved by the FDA for VHLD based on results from a phase II trial assessing drug efficacy in VHLD patients with ccRCC [38]. In this trial, 49% of ccRCC patients had objective responses in their tumor. In addition, other VHLD-associated lesions, including hemangioblastomas [39], pancreatic lesions (neuroendocrine tumors and serous cystadenomas) [40], and advanced pheochromocytomas and paragangliomas [41] also responded to the drug. In the LITESPARK-004 trial, which assessed treatment-naïve VHLD patients with at least one measurable ccRCC, 67% (41/61) of participants with ccRCC had an objective response at 50 months of follow-up, with 7 (11%) having a complete response and 34 (56%) a partial response [42]. Off-label use in late-stage VHLD patients with multiple lesions showed similar promising results, delaying disease progression and reducing the need for surgery [43]. Given the negative impact of surgery on the quality of life of VHLD patients [44], these are valuable gains.

Belzutifan has also been FDA-approved for use in patients with advanced ccRCC previously treated with immune checkpoint inhibitors and anti-angiogenic drugs. Approval was based on the outcome of the LITESPARK-005 trial, which showed that belzutifan outperformed the mTOR inhibitor everolimus in this cohort [45, 46]. Additional trials that assessed belzutifan plus the VEGFR inhibitor cabozantinib as frontline therapy for advanced ccRCC, found clinical benefit [47]. An ongoing topic of considerable interest is the establishment of biomarkers that predict response to belzutifan. To this end, studies of the expression of candidate biomarkers in clinical specimens are being augmented by laboratory evaluations of HIF-2α signaling dependencies (e.g. [48, 49]), with the goal of identifying strong responders, and efficacious therapeutic combinations. Such combinations may be useful for VHLD patients. However, while yielding enormous clinical benefit to individuals with established tumors, belzutifan is not ideal for cancer prevention/interception in VHLD patients, as it caused anemia and fatigue in a high proportion of treated individuals on various trials or when used off-label, with some subjects experiencing severe hypoxia. Long-term tolerability of belzutifan treatment remains to be determined [50–52].

In 2025, casdatifan (previously AB521) was reported as a second-generation inhibitor of HIF-2α [53, 54], based on preclinical analyses in mice and pharmacodynamic assessment in humans (NCT05117554). This orally bioavailable compound was completely selective for HIF-2α versus HIF-1α, resulted in no cytotoxicity in mice, and inhibited HIF-2α-dependent transcription. In studies of human xenograft tumors in athymic or humanized (NSG-MHC I/II DKO) mice, AB521 combined effectively with cabozantinib and the human anti-PD-1 antibody, zimberelimab. Pharmacokinetic and pharmacodynamic assessments indicated promising stability (half-life of ~ 24 h in humans).

The ARC-20 study,reported in 2026, was a first dose expansion clinical trial of casdatifan in in individuals with refractory metastatic ccRCC. Adverse events were similar to those seen with belzutifan (primarily hypoxia and anemia) and manageable, and 35% of treated individuals experienced an objective response. Reduction of serum biomarkers of erythropoietin, and the presence of high levels of HIF-2α and a strong signature for HIF-2α-dependent transcription in tumors, were both positive biomarkers for response to the drug. This study also reported genomic profiling results for treated patients, based on whole exome sequencing of 54 tumors. 85% of the patients had pathogenic single nucleotide variants and/or copy number loss in VHL; interestingly, neither VHL mutations or biallelic loss of VHL were predictive of response to casdatifan, distinguishing the consequences of VHL disruption from the elevation of HIF-2α and downstream transcriptional consequences.

### Additional biological activities and HIF-independent functions of VHL

Despite the historical focus on the VHL-HIF signaling axis, other VHL activities have been described, some of which do not depend on its association with and control of HIF proteins. Although the evidence for these VHL interactions and associated activities is strong, the mechanism by which disruption of these interactions by VHLD mutations contribute to the pathogenesis of the disease has attracted limited study.

#### VHL regulation of microtubules throughout cell cycle

**Fig. 4:**
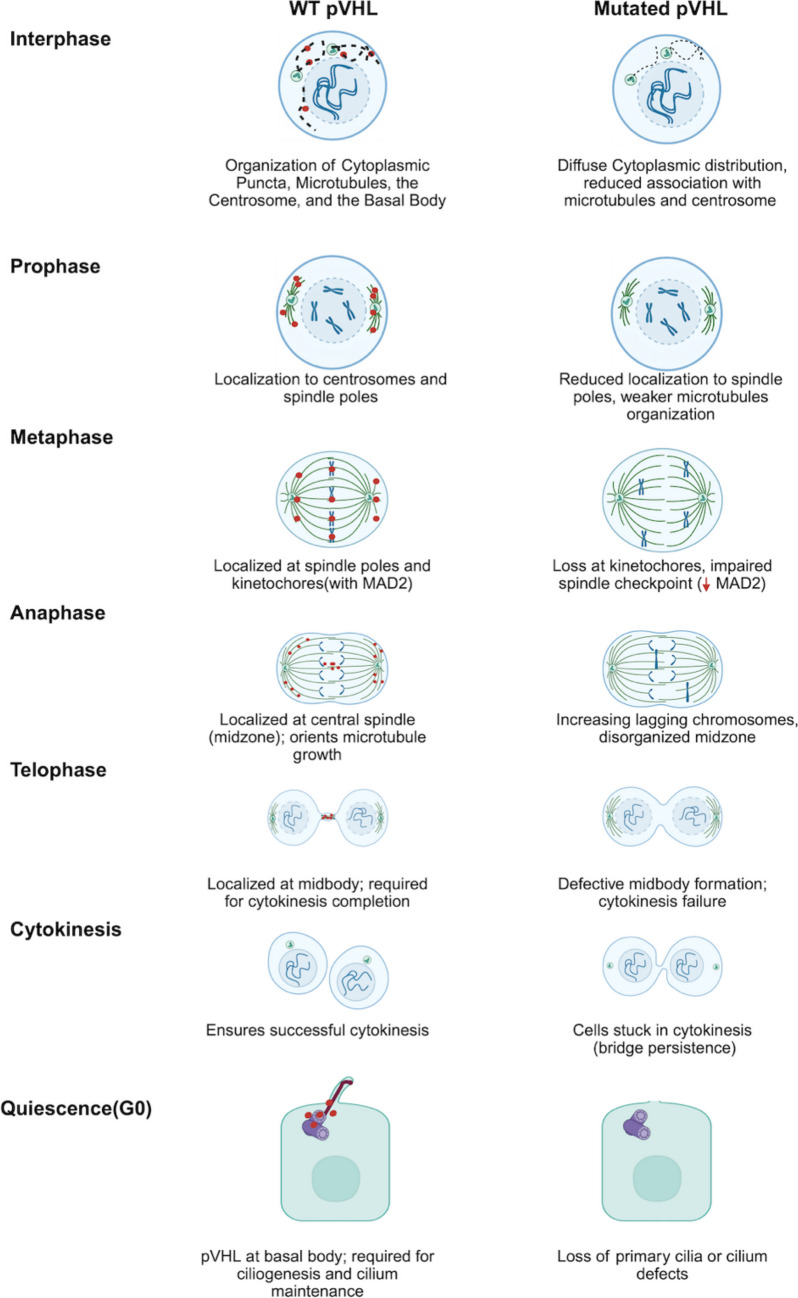
Non-canonical pVHL function in regulation of mitosis. **A**. The VHL protein associates with microtubules in interphase and mitotic cells; loss of VHL causes disorganization and destabilization of microtubule networks, and multiple mitotic defects, including chromosome mis-segregation, failure to complete mitosis, and altered plane of mitotic division; and loss of ciliation, (in G0 cells.)

There is strong evidence for VHL regulation of the microtubule cytoskeleton, with impact on cell polarity, and other processes (Fig. 4). An early study defined a microtubule-binding activity of pVHL, that depends on phosphorylation of VHL by GSKβ and contributes to stabilization of microtubule networks [55–57]. pVHL localizes to the mitotic spindle, and helps prevent spindle microtubules from disassembly induced by nocodazole. This function is selectively lost in cells expressing VHL mutations associated with type 2 A VHLD, which predisposes individuals to haemangioblastoma and phaeochromocytoma, but not to ccRCC [58]. In addition, inactivation of pVHL has been reported to induce spindle misorientation, spindle checkpoint weakening, and chromosomal instability, with associated aneuploidy. Such defects promote tumorigenesis and the formation of renal cysts (another feature of some forms of VHLD) [58, 59]. In a separate mitotic defect, loss of pVHL in ccRCC cells caused failure of cytokinesis, based on altered localization of factors required to resolve midbodies at the end of mitosis [60]. As a post-mitotic cell cycle defect, pVHL inactivation also causes loss of the primary cilium [61, 62], an antenna-like structure with a microtubule-based core that emerges from the basal body (derived from the centrosome) in G0 cells). Since cilia mediate mechanosensation and signaling, loss of cilia is strongly linked to defects in cell polarity, cyst formation, and cell cycle defects [63]. Altered ciliation and ciliary signaling can also influence tumor formation [64].

#### VHL signaling relevant to to cell cycle

Mechanistically, loss of pVHL has been reported to reduce expression of the mitotic checkpoint inhibitor MAD2 [58], while elevating expression of a distinct ubiquitination substrate and mitotic checkpoint regulator MPS1 [65]. VHL loss induces the transcription of AURKA, a kinase regulating mitotic entry and spindle integrity, and its partner and activator HEF1/NEDD9 [66–68]. AURKA has also been defined as a direct VHL substrate, with pathogenic VHL mutations elevating AURKA by decreasing its degradation [69]. VHLD-associated defects in the regulation of microtubules [70] and subsequent elevated AURKA expression [71] also contribute to the loss of ciliation; ciliary loss is associated with cystogenesis and RCC. VHL regulation of ciliation is also impacted by additional effectors, including HIF-dependent control of the ciliary kinase NEK8 [72], and other mechanisms.

#### VHL signaling and resistance to cell death

In addition to mitotic controls, pVHL regulates directly influences cell survival through non-HIF-dependent signaling, via hypoxia-dependent or -independent mechanisms. A number of pVHL-regulated proteins influence cell survival. pVHL directly binds the pro-survival kinase AKT, following its proline-hydroxylation under hypoxic conditions, inhibiting its activity [73]. pVHL also suppresses the activity of another pro-survival factor, NF-κB, by serving as a physical bridge between the NF-κB agonist CARD9 and CK2, increasing CK2-dependent inhibitory phosphorylation of CARD9 [74]. As an alternative way of controlling NF-κB, VHL additionally binds and negatively regulates the transcription factor ZHX2, which promotes NF-κB activation [75]. VHL binds EGLN3-hydroxylated BIM-EL, a pro-apoptotic protein, inhibiting the phosphorylation of BIM-EL by ERK, and thus promoting its degradation; mutations associated with type 2 C VHLD eliminate this ability [76]. A type 2 C mutation (pVHL L188V, affecting residues buried in the hydrophobic core of the α-domain) has also been shown to retaining ability to bind and regulate HIF-2α, but lose the ability to promote assembly of an extracellular fibronectin matrix (ECM) [77], previously reported as an important activity of pVHL [78]. As fibronectin signaling through integrins plays a major role in regulation of cell survival, differentiation, and migration, dysregulation of this property could contribute to type 2 C pathogenesis.

In some cases, it is difficult to separate *VHL* mutation phenotypes from the regulation of HIF proteins. As an example, the atypical protein kinases a PKCλ and aPKCζ, which signal through phospholipid effectors to regulate cell survival, proliferation, polarity, and differentiation, bind directly to pVHL [79]; VHL binding to aPKCλ was found to lead to its ubiquitination and destruction [80]. The site of interaction of with VHL is in a region of the β-domain that overlaps with the HIF-2α binding site, and is the site of missense mutations leading to Types 1 and 2 C of VHLD [79]. Hence, mutations disrupting VHL disruption of HIF signaling will simultaneously disrupt control of these aPKCs – and potentially other pVHL substrates.

#### VHL and epigenetic control of gene expression

Beyond targeting transcription factors such as HIF and ZHX2, pVHL also directly regulates gene expression through additional mechanisms. For example, pVHL binds the H3K9 methyltransferase and chromatin regulator SETDB1 in an oxygen-dependent manner, triggering its degradation; hypoxia or loss of pVHL elevates expression of SETDB1. An important function of SETDB1 is to repress expression of transposable elements, which both trigger genomic instability and an immune-inflammatory response [81]. How SETDB1 functions to influence the growth properties of tumors with mutated VHL has not been investigated. pVHL also can target subunits of RNA polymerase, affecting the stability of the core transcriptional apparatus [82]. As another example, N6-methyladenosine on RNA (m6A) post-transcriptionally regulates gene expression, influencing the cellular decision to translate or degrade specific mRNAs; m6A modifications are dysregulated in ccRCC. pVHL directly targets the METTL3/METTL14 complex that mediate the addition of m6A to mRNAs, with one recent study identifying relevant targets such as PIK3R3 that control activation of the pro-survival factor AKT [83]. Additional HIF-independent functions are discussed in [84, 85].

#### Post-translational modifications of VHL identify additional functional dependencies

A number of post-translational modifications of pVHL have been reported, including sites of phosphorylation, sumoylation, and ubiquitination (summarized at www.phosphosite.org). Kinases that have been identified as modifying pVHL include AURKA (on S72, [86]), CDK1 (on S80, [87]), CHK2 (on S111, [88]), GSK3β (on S68, [56]), as well as NEK1 [89] and casein kinases [90] on numerous residues. These phosphorylation events have been reported to affect pVHL stability and protein interactions. Hypoxic stress has been shown to induce sumoylation of pVHL on K171 and K196, resulting in VHL oligomerization and increased stability, but also reducing its ability to bind HIF proteins [91]. Several studies have reported neddylation of pVHL at K159 based on activity of NEDD8 [92] and MDM2 [93]. This modification disrupts interactions of pVHL with CUL2, and has been described as a molecular switch, altering the biological activity of pVHL to E3-ligase independent activities (e.g., such as promoting fibronectin matrix assembly [94]). However, for many of the identified post-translational modifications, functional roles in cancer risk for VHLD patients remain poorly defined.

### Targeting pVHL for VHLD and ccRCC

Given that *VHL* mutations are the basis for VHLD, drugs selectively restoring the function of pVHL impaired by disease-associated missense mutations would theoretically represent optimal agents for interception of VHLD-associated pathological conditions. Depending on the nature of the missense mutation, pVHL-targeted compounds could work either by restoring a weakened interaction between pVHL and the elongin complex; strengthening a weakened interaction between pVHL and HIF; or helping to refold or stabilize expression of pVHL, in cases where missense mutations reduce the expression level of appropriately folded VHL protein. Use of the proteasomal inhibitors bortezomib and carfilzomib have been shown to increase the endogenous levels of specific destabilizing VHL missense mutations [95]. The chaperone HSP90 has been shown to contribute to pVHL degradation [96]. SAHA and LB-205—histone deacetylase inhibitors (HDACis) that modulate the binding of chaperones such as HSP90 to pVHL – have been shown to significantly increase the half-life of pVHL with destabilizing mutations [97]. Besides blocking HSP90 interactions with pVHL affected by a number of distinct germline mutations, treatment with the HDACi vorinostat increased pVHL expression and suppressed the transcription of HIF-dependent transcripts [98]. Further, a clinical trial using vorinostat (NCT02108002) has identified promising biological activity in VHLD patients with central nervous system hemangioblastomas prior to surgical removal. However, none of these broadly-acting compounds are suitable for use in VHLD, due to toxicities associated with sustained use.

Others have used high affinity VHL-binding tool compounds to explore VHL pathway activity [99]. Among targeted compounds, VH298 is the most potent member of a compound series that was identified initially with the goal of disrupting the pVHL-HIF interaction interface [100–102]. VH298 was designed as a HIF mimetic and competitor for HIF binding. Structural analysis of the compound bound to the VCB complex defined its binding site as including residues D67, R69, W88, F91, W98, P99, R107, H110, S111, Y112 and H115 in the N-terminal β-domain [100]. Initial experiments with this compound and analogs (e.g. VH032) demonstrated activity in inducing a subset HIF-dependent transcripts, although with a spectrum of transcripts distinct from that observed for hypoxia or a prolylhydroxylase domain inhibitor (PHDi), FG-4592 [102]. Several studies indicate VH298 is well tolerated at high levels, and exhibits biological activity in a variety of disease settings in rodent models, including promotion of bone-tendon connections following damage [103], and wound healing in hyperglycaemic rats [104]. VH298 has also been linked to other cellular processes, such as pexophagy (autophagy of peroxisomes), behaving similarly to an inhibitor of HIF [105]; and as an inducer of Th17 immune cell differentiation [106]. All activities were associated with elevated HIF-dependent transcription.

A more recent and surprising finding with VH298 indicated a more nuanced activity [107]. While short-term administration of VH298 induced expression of prolyl-hydroxylated HIF, as predicted, extended VH298 treatment caused an increase in pVHL protein expression based on an as yet undefined, post-transcriptional mechanism. This delayed increase in pVHL expression was sufficient to lead to suppression of HIF [107]. This bimodal response complicates interpretation of *in vivo* findings with this compound. Further, as discussed below, pVHL serves as a substrate recognition component for degradation of a significant number of proteins. The interface used by pVHL to interact with HIF interface also mediates pVHL interaction with other proteins, although the structural elements of these interactions are much less well defined. Although it is likely that VH298 is disrupting some or many of these additional interactions, it is unclear how such altered interactions contribute to the observed effects of VH298.. Further, given that VHLD-associated missense mutations span the protein sequence and have few hotspots, it is plausible that mutations targeting this interface may disrupt the interaction of pVHL with more than just HIF. Further progress will depend on a more granular understanding of VHL protein interactions, and structure-interaction-functional relationships; a topic of intense interest in the field.

### Location and functional consequences of VHL mutations associated with distinct classes of VHLD

As more families with germline mutations in VHL were identified, it soon became clear that there was a broad spectrum of disease phenotypes associated with VHLD. Aligning the genotype–phenotype relations of specific VHLD-associated mutations has been a topic of interest for over three decades [108–110]. A large number of VHLD mutations have been compiled in publicly accessible databases, with some recent publications detailing national registries of VHLD patients [3, 111–119]. More recent studies have used *in silico* analysis and machine learning to probe the structural and functional consequences of large numbers of naturally occurring mutations [3, 117]. Aligning the location and type of mutations together with known subtypes of VHLD (Table 1) has led to several conclusions:

**Table 1:** Subtypes of VHL disease, and associated mutations

VHL Class	Renal Cell Carcinoma	Hemangioblastoma	Paragangliomas	Pheochromocytoma	Predominant Mutation Type
Type 1	High	High	Rare or absent	Rare or absent	Large deletions, nonsense, splicing mutations, non-stop mutations. Target α and β domains; missense mutations often destabilize protein (buried residues), or disrupt interface with HIF or elongins
Type 2A	Rare	High	Rare or absent	High	Missense; target surface exposed residues; do not necessarily disrupt HIF or elongin interactions
Type 2B	High	High	Medium	High	Large deletions, nonsense, splicing mutations, non-stop mutations. Target α and β domains; missense mutations often destabilize protein (buried residues), or disrupt interface with HIF or elongins
Type2C (uncommon)	Absent	Absent	High	High	Missense; target surface exposed residues; do not necessarily disrupt HIF or elongin interactions

First, the majority of germline mutations that result in a pathogenic phenotype fall within the structured α and β domains of pVHL, with almost none identified within the N-terminal sequences absent in pVHL19.

Second, missense mutations associated with moderate to high levels of ccRCC (Type 1 and 2B VHLD, with high likelihood of ccRCC and hemangioblastomas) are more likely to affect buried residues that destabilize the protein than those associated with other forms of VHLD. Similarly, the highest frequency of nonsense and splicing mutations, and non-stop mutations and deletions that lead to protein loss, are found in Type 1 and 2B VHLD. Alternatively, ccRCC-associated mutations target the interfaces between pVHL and either elongin C or HIFα, and eliminate binding to these proteins. Collectively, such mutations lead to the highest levels of HIF induction. Interestingly, phenotypes of VHLD can also arise from inherited mutations affecting critical interacting partners such as elongin C (ELOC), which disrupt the pVHL-ELOC interface [120].

Third, many mutations associated with ccRCC and strong HIF induction result in destabilization of the VHL mRNA, indicating the loss of VHL function occurs by multiple mechanisms.

Fourth, some VHLD types that lead to paragangliomas and pheochromocytomas, but not ccRCC (Type 2 C VHLD, and some Type 2B), typically have little if any induction of HIF. These mutations are more likely to target surface-exposed residues on VHL. For these tumors, it is hypothesized that alternative VHL interactions that are affected by VHL mutations associated with Type 2B/C VHLD. One study of type 2B/C variants indicated that these mutated forms of VHLD were associated with decreased expression of the 2-oxoglutarate–dependent oxygenase EGLN3, reducing apoptosis in neural crest cells [121]. Further work indicated that the EGLN3-dependent hydroxylation of the pro-apoptotic protein BIM-EL normally promoted direct interaction with VHL, and stabilized BIM-EL; however, 2C-class VHLD mutations were unable to interact with BIM-EL, leading to its destabilization and increasing the survival of neural cells [76]. At present, it is unclear whether BIM-EL is the sole critical target of VHL affected in Type 2 C VHLD, or whether additional targets exist.

Fifth, the disease specificity of specific VHLD mutations is likely to reflect negative as well as positive selection pressures. While hyperactivation of HIF-dependent transcription is essential for ccRCC, excessive HIF activity is lethal in many other cell types, including neuroblastomas and melanomas; only moderate elevation of HIF activity is suitable for promotion of paragangliomas and pancreatic neuroendocrine tumors [122–125].

Sixth, a very large number of proteins have been proposed to interact with VHL, with many captured in an online database (http://vhldb.org, [112]). Some of these interactions are known to involve the same pVHL domains required for interactions with elongins or HIFα. While the strength of evidence supporting these interactions varies, numerous HIF-independent activities of VHL clearly exist, and some subtypes of VHLD must involve loss of non-HIF and/or non-elongin interactions. Even for Type 1 VHLD, which clearly depends on loss of interactions that result in the degradation of HIF, it is possible that differential response to belzutifan among distinct patients, and the overall spectrum of possible VHLD-associated phenotypes that are experienced by individuals with a given germline VHL variant, reflects differing subsets of interactions that are disrupted by the same variant. However, given that the structural features of most non-HIF interactions are poorly defined, the effect of specific mutations on the broader interaction landscape of pVHL requires intensive future study.

Seventh; the lack of detailed knowledge about the interface between VHL and its interactive partners affects the current ability to design combination or alternative therapies. For example, for some pVHL-interacting proteins, including AURKA, AKT, aPKCs, and a number of others, inhibitory compounds exist and have undergone clinical testing. Depending on the binding site of these proteins on pVHL, such compounds may have activity with only a subset of VHLD-associated mutations, and the activity of multiple partners may be affected by a single mutation. Given the heterogeneity of VHL mutations that exist in the population, it will be necessary to develop robust biomarkers for the relevance of individual partners in the context of distinct VHL mutations, as well as genetically defined cohorts that might yield a positive signal from administration of an inhibitor of a partner protein in a clinical setting.

### Developing therapies for VHLD; future prospects

The availability of HIF-targeting agents such as belzutifan has greatly improved the tools for management of established tumors in individuals with VHLD-associated or sporadic ccRCC. A current topic of investigation is how to increase the primary response rate to HIF-targeting agents, or to prevent or overcome acquired resistance. To this end, investigations of synthetic lethality relationships with VHL loss [126–129], or exploiting downstream consequences of VHL deficiency [130, 131], can be useful in suggesting combination therapies. One limit on these approaches has been the failure, to date, to develop a VHLD (or any) mouse model that forms kidney cancers with HIF dependence [132], which has made it difficult to test concepts for combination therapy in a preclinical setting.

Management of a genetic disease such as VHLD, including development of effective therapeutics to prevent, intercept, or treat associated cancers, is fundamentally different from treatment of cancers with sporadic pVHL mutations. Individuals will be heterozygous for a damaged VHL allele; potential preventive agents must not trigger excessive cytotoxicity in a haploinsufficient setting. Therapies for prevention or interception must be safe when applied over a period of years. Multiple primary tumors will emerge over time, based on loss of the wild type copy of VHL; surveillance throughout the lifetime is critical.

Given the well-defined, monogenic and dominant nature of mutations leading to VHLD, future management may include the use of preimplementation genetic testing (PGT); this approach is now being used in some cases to eliminate other cancer-predisposing variants from familial germlines [133, 134]. Immune checkpoint inhibitors have yielded positive outcomes in many cases of ccRCC [8], and recent findings that HIF upregulates endogenous retroviruses (ERVs) [135] suggest a potential new class of target for immunotherapies. However, the toxic side-effects of such agents will limit their use in individuals with type I VHLD who are prone to the repeated development of primary renal tumors. One promising approach for spontaneous ccRCC has been the development of personalized cancer vaccines (PCVs) to neoantigens associated not only with mutated, driver VHL, but also other known driver oncogenes for ccRCC (PBRM1, BAP1, KDM5C and PIK3CA) [136]. While this approach resulted in the development of durable T cell clones reactive against the tumor, such an approach would need to be adapted for VHLD patients, given that in individuals born with pathogenic VHL alleles, every cell in their bodies bears the defective gene, excluding direct targeting of mutated VHL. Nevertheless, a recent study with Lynch Syndrome patients demonstrates the promise of a neoantigen-targeted PCV approach [137], supporting investigation in this direction.

While PCVs may not be appropriate, future treatment of VHLD may increasingly incorporate personalized analysis of the impact of specific germline variants unique to individuals or families. Depending on the specific variant, the encoded mutant pVHL may be poorly expressed or rapidly degraded, causing some degree of haploinsufficiency. Alternatively, if a mutated form of pVHL persists, it may potentially acquire neomorphic functions associated with an altered protein interaction spectrum that differs for unique variants. The advent of sophisticated artificial intelligence-based platforms for protein structural modeling such as Alphafold3 [138] should assist in assessing the impact of specific mutations on interactions between pVHL and partners. For VHLD families and individuals, contact with international societies that maintain current information about clinical management will help in decision-making for optimal care. Among these, the VHL Alliance (http://vhl.org) is a valuable resource.

Inherited cancer syndromes have long been considered particularly challenging to manage. With the rapid advances in studies of pVHL, better surveillance and screening, the emergence of new technologies to modulate gene expression and protein function, and breakthroughs in gene editing to repair damaged alleles, there is hope that curative options may emerge for VHLD patients in the near future.

## Data Availability

No datasets were generated or analysed during the current study.
